# The link between selection for function and human-directed play behaviour in dogs

**DOI:** 10.1098/rsbl.2020.0366

**Published:** 2020-09-23

**Authors:** Niclas Kolm, Hans Temrin, Ádám Miklósi, Enikő Kubinyi, László Zsolt Garamszegi

**Affiliations:** 1 Department of Zoology, Stockholm University, Svante Arrhenius väg 18B, 10691 Stockholm, Sweden; 2 Department of Ethology, ELTE Eötvös Loránd University, Budapest, Hungary; 3 MTA-ELTE Comparative Ethology Research Group, Budapest, Hungary; 4 Institute of Ecology and Botany, Centre for Ecological Research, Vácrátót, Hungary; 5 MTA-ELTE, Theoretical Biology and Evolutionary Ecology Research Group, Department of Plant Systematics, Ecology and Theoretical Biology, Eötvös Loránd University, Budapest, Hungary

**Keywords:** dogs, artificial selection, play, domestication

## Abstract

Human-directed play behaviour is a distinct behavioural feature of domestic dogs. But the role that artificial selection for contemporary dog breeds has played for human-directed play behaviour remains elusive. Here, we investigate how human-directed play behaviour has evolved in relation to the selection for different functions, considering processes of shared ancestry and gene flow among the different breeds. We use the American Kennel Club (AKC) breed group categorization to reflect the major functional differences and combine this with observational data on human-directed play behaviour for over 132 breeds across 89 352 individuals from the Swedish Dog Mentality Assessment project. Our analyses demonstrate that ancestor dogs already showed intermediate levels of human-directed play behaviour, levels that are shared with several modern breed types. Herding and Sporting breeds display higher levels of human-directed play behaviour, statistically distinguishable from Non-sporting and Toy breeds. Our results suggest that human-directed play behaviour played a role in the early domestication of dogs and that subsequent artificial selection for function has been important for contemporary variation in a behavioural phenotype mediating the social bond with humans.

## 1. Introduction

Play behaviour is a near-ubiquitous aspect of sub-adult behaviour in mammals (e.g. [1]). The potential adaptive value of play behaviour is broad, including positive effects on the development of both physical and social abilities in young individuals (e.g. [2–4]). In general, it is uncommon that animal play behaviour extends to adulthood, although some examples also exist for play behaviour occurring among individuals of mixed age-classes (e.g. [5,6]). Also, although examples exist (e.g. [7,8]), it is relatively uncommon that play behaviour extends to interspecific interactions. Such phenomena are most often found in captive or domesticated species [1]. One such example is the domestic dog, a species that spends substantial amounts of energy and resources on human-directed play behaviour (e.g. [9–11]). The domestic dog also differs from its ancestral species, the wolf, in that play behaviour and in particular human-directed play behaviour is much more frequent and maintained throughout adulthood. For instance, in a recent comparison between dogs and wolf–dog hybrids, dogs were much more likely to engage in playing with human playmates [12].

It has been suggested that human-directed play behaviour was important during dog domestication and dog breeding in general, and that selection for particularly playful individuals may have played an important role in the artificial selection regime that the domestic dog has gone through in the past few hundred years [9]. This could have been particularly important in working breeds, where accentuated human-directed play behaviour may have been an important training tool that also strengthened the social bond between for instance a hunter and its hunting dog (e.g. [9,13]). However, until now, no broad-scale analysis exists on human-directed play behaviour evolution in the domestic dog in light of recent insights into the genetic relationships between different dog breeds (e.g. [14]). Here, we provide such a study by using a large dataset on behaviour across all major dog breed functions to investigate how human-directed play behaviour has evolved in relation to artificial selection for breed function. All analyses are based on an analytical approach that controls for effects of shared ancestry and gene flow among the different breeds [15].

## 2. Methods

### (a) Data collection

The behavioural data originated from the Dog Mentality Assessment (DMA) project of the Swedish Kennel Club, which arranges standardized behavioural tests on purebred dogs provided voluntarily by owners since 1989 [16]. Specifically, we used data corresponding to a period of 17 years (1997–2013), during which information on 89 352 individual dogs from 138 breeds was collected (electronic supplementary material), of which 132 are categorized by the American Kennel Club (AKC). Information on genetic relatedness from Parker *et al*. [14] was also available for the included breeds. All dogs were older than 1 year of age. DMA relies on a strict protocol, where behavioural scores are assigned to the subjects in a fixed set of test situations to characterize various behavioural aspects [16]. We focused on the assessment of behaviour that describes the dog's interest to play with humans. In this test, the owner initiates playing with the unleashed dog by providing a toy in the shape of a piece of rag. Once the dog accepts the invitation to play, the owner throws the rag to the test leader (stranger), and then the rag is thrown twice between the test leader and the owner before the test leader throws the rag away, allowing the dog an opportunity to take it. Then, the owner gently takes the rag away from the dog and hands it over to the test leader who holds the rag with both hands and invites the dog to grab it for a ‘tug of war'. Based on the reactions of the subject dog in this owner/stranger playing context, it is given one of the following scores: (i) does not play and shows no interest in the tossing of the rag, (ii) does not play but shows interest, (iii) plays, but only after a slow start, (iv) plays actively after a fast start, (v) starts very fast, and plays very actively. Based on these observations, we calculated the mean of scores for each breed and treated these as breed-specific estimates of human-directed play behaviour. See Garamszegi *et al*. [15] for detailed information on the considerable levels of repeatability and reliability of these mean scores. Importantly, human-mediated play behaviour towards the stranger in the DMA protocol strongly correlates with play behaviour towards the owner [15]. We present additional analyses to show that within-individual effects do not confound breed-specific estimates of the mean and variance of play behaviour in the electronic supplementary material.

As a proxy for the artificial selection regimes acting on the function of ancestors of modern breeds, we categorized each dog breed in our dataset according to the seven main functional groups recognized by AKC (www.akc.org). These include the Herding Group (*n* = 20, breeds used for their ability to control the movement of other animals), the Hound Group (*n* = 17, breeds used mainly for hunting in conspecific groups), the Toy Group (*n* = 14, breeds with the small size used for companionship), the Non-sporting Group (*n* = 17, breeds used as watchdogs and housedogs with substantial variation in behaviour and morphological features), the Sporting Group (*n* = 19, breeds used during hunting to locate or retrieve quarry), the Terrier Group (*n* = 19, breeds used for hunting, vermin control, and guarding) and the Working Group (*n* = 26, often large breeds used for working duties such as guarding and pulling sleds). See electronic supplementary material for a full description of the functional groups and dataset.

### (b) Analysis

To investigate how artificial selection for particular functions was distributed along the phylogeny (phylogeny was extracted from Parker *et al*. [14]), we first performed an ancestral state reconstruction of the AKC groups. We used the *ace* function of the R package ‘ape' [17], which estimates ancestral character states (i.e. the chance of the ancestor of all breeds belonging to any specific functional group) and the associated uncertainty for discrete characters based on maximum likelihood [18]. We assumed an evolutionary model based on equal-rates (ER) transitions, but when we used other models the results were qualitatively very similar.

We also reconstructed the ancestral states (including their 95% confidence intervals) for the continuous focal trait, human-directed play behaviour based on maximum likelihood [19]. We used the *fastAnc* function of the R package ‘phytools' [20] assuming the default random walk Brownian motion model. To characterize phylogenetic signal in play behaviour (i.e. to see how closely related breeds share the same phenotype), we used the *fitContinuous* function of the R package ‘geiger' [21] to estimate Pagel's lambda [22] along the scale of 0–1 (where 1 indicates that the phylogeny perfectly predicts covariance among the trait values of breeds, and 0 indicates no phylogenetic structure in the data at all).

To test the relationship between human-directed play behaviour and AKC groups, we followed a framework that allows partitioning among-breed level variances into a phylogenetic component and into a component that reflects gene flow [23]. A control for gene flow might be important for dogs because crosses among breeds may raise similarity in the focal phenotype in addition to constraints due to common descent. We have previously shown that the confounding effect of admixture due to the crosses of different breeds is relatively small for human-directed play behaviour in comparison to the effect of genetic distance reflecting the phylogenetic history of breeds [15]. However, to ensure ample control for any variance caused by gene flow, we constructed a phylogenetic mixed model that included the genetic similarity matrix and haplotype sharing matrix as random effects to account for common ancestry and gene flow in the evolution of human-directed play behaviour. This model was fitted using the ‘MCMCglmm' package [24] with AKC groupings as the main predictor and also enabled us to take into account within-breed variance in the focal behaviour in the form of measurement error variance. For further detail on these analyses, see electronic supplementary material and Garamszegi *et al*. [15].

## 3. Results

**Figure 1.**
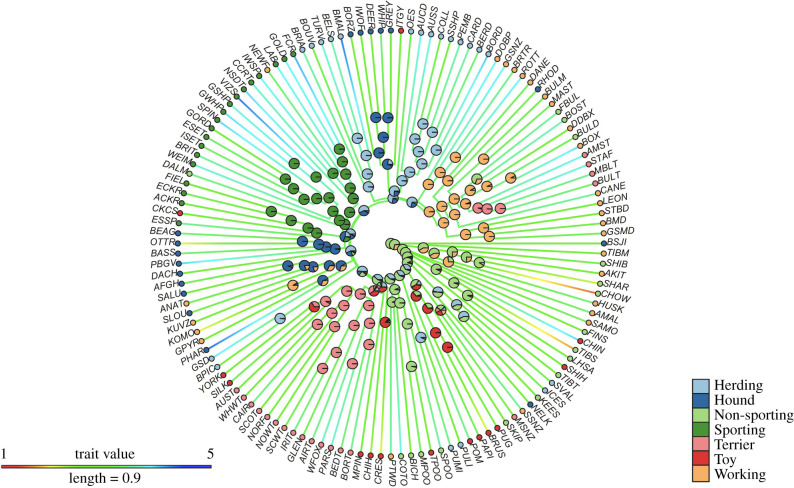
Ancestral state reconstruction of AKC (American Kennel Club) categorization of breeds and human-directed play behavior across breeds. Pies at the nodes of the phylogeny indicate scaled likelihoods for particular AKC categories. At the root of the phylogeny, these likelihoods are Non-sporting: 0.68; Working: 0.27; Hound: 0.02; Herding: 0.01; Toy: 0.008; Terrier: 0.003; Sporting: 0.003. For the reconstruction of human-directed play behaviour scores, the trait value is colour coded along the branches of the phylogeny such that red colour indicates the lowest levels of play behavior while blue colour indicates the highest levels of play behaviour. Breed abbreviations are listed at the tips of the phylogeny (see electronic supplementary material for corresponding breed names).

The breeds clustered on the phylogeny in accordance with the AKC groupings (figure 1). Partly intermixed with Working (scaled likelihood value at the root: 0.27), the Non-sporting group (scaled likelihood: 0.68) represented the most likely ancestral function in the phylogeny (scaled likelihood values for other groups less than 0.05), followed by an increased probability of Herding along with the descendant nodes.

**Figure 2.**
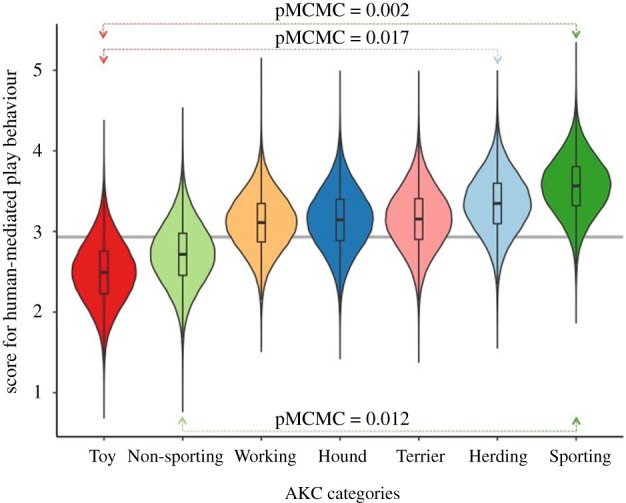
The posterior distribution of human-directed play behaviour in different functional groups of breeds as reflected by AKC categorization. The violin plots show the probability densities at different values reflecting the frequency by which the MCMC chain sampled them under the specified phylogenetic mixed model accounting for effects due to shared ancestry and gene flow (table 1). The boxplots inside the violins depict the ranges of the posterior distributions as well as their lower hinge, median and upper hinge. Coloured arrows indicate post hoc comparisons with significant differences of the corresponding posterior distributions in table 1 (pMCMC less than 0.05). The grey horizontal line reflects the estimate of the mean ancestral state of the trait for illustration.

Our ancestral state reconstruction revealed intermediate ancestral levels of human-directed play behaviour (ancestral state score = 2.93, 95% CI = 2.57–3.29; figures 1 and 2, from which the trait evolved in a phylogenetically constrained fashion (lambda = 1).

**Table 1.** Random and fixed effects of a random intercept linear mixed model partitioning the components of among-breed variance of human-directed play behaviour in dogs due to common ancestry and gene flow as random effects and evaluating the importance of AKC categorization reflecting selection for function as fixed predictor. The analysis relies on breed-specific mean estimates of human-directed play behaviour while accounting for the intra-breed variance.

random effects	*σ*^2^ (95% CrI)
genetic distance (common ancestry, $\sigma_{a}^{2}$)	0.354 (0.071–0.765)
haplotype sharing (gene flow, $\sigma_{b}^{2}$)	0.133 (0.056–0.222)
residual ($\sigma_{e}^{2}$)	0.012 (0.001–0.043)
fixed effects	*β* (95% CrI)
AKC categorization (Herding)	3.341 (2.603–4.074)
AKC categorization (Hound)	3.142 (2.386–3.883)
AKC categorization (Non-sporting)	2.709 (1.930–3.456)
AKC categorization (Sporting)	3.553 (2.801–4.221)
AKC categorization (Terrier)	3.152 (2.413–3.889)
AKC categorization (Toy)	2.491 (1.748–3.279)
AKC categorization (Working)	3.101 (2.385–3.768)

For the comparison of human-directed play behaviour scores across the AKC groups, we compared the posterior distribution of human-directed play behaviour separately in each functional group as calculated from the appropriate phylogenetic mixed model summarized in table 1 (this model had a DIC value that was 13.6 units lower than that of the null model only including the intercept). This comparison revealed that the Herding (3.51) and Sporting (3.71) Groups had the highest levels of human-directed play behaviour and that these were significantly different from the Non-sporting and Toy Groups (figure 2 and table 1).

## 4. Discussion

The estimate for the common ancestor of the dog breeds included in the study shows intermediate levels of human-directed play behaviour. This is the first formal comparative analysis showing this pattern, and it supports the idea that human-directed play behaviour could have been an important trait during dog domestication, as suggested by several previous studies [9,12,25]. The strong phylogenetic signal in both human-directed play behaviour and the AKC functional breed groups further suggests that the selective protocol for the different breeds has quite effectively separated the AKC breed groups for these traits. This matches previous results from clustering analyses of behavioural traits in dogs (e.g. [26,27]).

The association between functional group type and human-directed play behaviour supports that artificial selection of specific breed qualities has also affected human-directed play behaviour. This result differs from a previous study by Svartberg [13] that did not detect differences in playfulness among different breed types. We suggest that the smaller sample size and the lack of control for shared ancestry in the previous study explain this difference.

Our observed difference across breed types could be explained by at least two mechanisms. First, other important traits that were targeted during the breed type selection regimes could be genetically correlated with human-directed play behaviour causing correlated differences in human-directed play behaviour between the breed types. Different behavioural traits are often strongly correlated during domestication of animals, and this is the case also in dogs [28–30]. However, previous evidence suggests that play behaviour in the human–dog context is relatively independent of play behaviour in the dog–dog context [31]. Moreover, a recent study demonstrated surprisingly weak correlations between different behavioural traits (including human-directed play behaviour) in modern dog breeds, while more ancient breeds displayed stronger behavioural correlations [32]. Hence, human-directed play behaviour appears to be relatively independent of other behavioural traits, at least in modern breeds, and it is thus less likely that our discovered pattern stems only from other traits under selection being linked with human-directed play behaviour.

Second, human-directed play behaviour could itself be an important trait under direct selection during the breed function selection regimes. Closer examination of the groups with the highest scores in human-directed play behaviour, the Herding and Sporting Groups in the AKC grouping system (electronic supplementary material), shows that these groups include dog breeds that work in close cooperation and continuous visual contact with their human handler, such as retrievers, pointers and collies [33]. The functions of these breeds, for instance retrieving prey or herding livestock, build on an appropriate social relationship and strong bond with the handler, aspects that could be strengthened with frequent play. This further supports the idea that human-directed play behaviour was itself an important trait in the breeding of these dogs [9]. In a previous study on behavioural variation among AKC functional groups, Turcsán *et al*. [26] showed that scores of trainability, another behavioural trait of interest during dog breeding in this context, were also relatively high in the Herding and Sporting Groups. In their study, the factor scores of trainability partly built on aspects of play behaviour [26] making it difficult to disentangle the two traits. But put together, these two studies certainly point towards the possibility of a general evolutionary link between breed function, trainability and human-directed play behaviour during dog breeding.

## Supplementary Material

Supplemental Material on analyses and breed function group descriptions

## Supplementary Material

Complete database with breed abbreviations and scripts

## Data accessibility

See electronic supplementary material and attached Excel sheet for a full description of the functional groups and dataset.
