# Supplementary material for: The link between selection for function and human-directed play behaviour in dogs
Source: Biol Lett. 2020 Sep 23;16(9):20200366. doi: 10.1098/rsbl.2020.0366 (PMC7532715; doi:10.1098/rsbl.2020.0366)
Supplement: Supplemental Material on analyses and breed function group descriptions [file rsbl20200366supp1.docx]

**Supplemental Material**

**Assessing potential confounders at the within-breed level: the reliability of breed-specific values**

In a companion paper, we have performed several statistical analyses at the within-breed level in order to verify that the breed-specific estimates of human-mediated play behavior are biologically meaningful and can be reliably used in the among-breed analyses (Garamszegi et al. 2020). In particular, we investigated how different potentially confounding variables can influence the within-breed variance of the trait, and how these effects may bias the breed-specific mean trait estimate that we used in our phylogenetic comparative analyses as the focal unit.

As supporting information to this companion paper (Garamszegi et al. 2020), we presented the methods, results and the appropriate interpretation of a linear mixed modeling (LMM) exercise that was used to study the effect of different confounders at the within-breed level. In this model, we entered the individual-specific, raw scores of play behavior as response variable by assuming normally distributed errors, while the following potentially confounding factors were added as fixed predictors: sex, age (but only individuals that were older than 1 year were considered), year of testing (to deal with potential trend effects), and season (to deal with potential seasonal effects). Due to the hierarchical structure of the data, we also investigated the following variables as random effects: identity of the test-leader, identity of mother, identity of father, year of testing (as factor) and breed identity. The model revealed that both the investigated fixed and random terms are important effects mediating differences among individual dogs in how they play with humans (see Table S1 of Garamszegi et al. 2020).

From this model, we extracted the best linear unbiased predictors (BLUPs) as estimates of the breed-specific scores of human-directed play behavior when controlling for the variables that were included in the LMM. Therefore, by assuming that these BLUPs account for the confounding effect at the within-breed level, we examined how these corrected estimates support the breed-specific mean estimates of the scores (that neglect within-breed effects in essence). We found that these two sets of estimates strongly correlate with each other (*r* = 0.962, *N* = 138, *P* < 0.001). Hence, we can reasonably conclude that the within-breed effects do not raise serious influences at the between-breed level, as these do not confound the breed-specific estimates of the mean of the focal variable. Note also that the breed-specific estimates as calculated from the DMA project strongly correlate with estimates of the same phenomenon from an independent source (Turcsán et al. 2011), which also confirms that the variable we used in our comparative analyses is biologically meaningful (Garamszegi et al. 2020).

There were some degrees of relatedness structure in the raw data, as for some breeds (especially in breeds with large sample size), there were some individuals that originated from the same dog family. The above model revealed non-negligible effects for maternal and paternal identities (i.e. ~ heritability) on scores of play behavior indicating that relatedness structure within breeds needs particular attention. Therefore, in addition to the approach based on BLUPs that account for paternal and maternal effects, we also investigated the effect of familiar relationship within breeds in a different way. Accordingly, by randomly picking one individual from each family, we removed related individuals from the raw database to diminish any inbreeding effect. Then, we recalculated the breed-specific means and variances for play behavior based on scores from unrelated individuals. These estimates strongly correlated with the original breed-specific estimates that were calculated by considering all available individuals irrespective to their familiar relationships (mean: *r* = 0.959, *N* = 138, *P* < 0.001, variance: *r* = 0.814, *N* = 135, *P* < 0.001). Therefore, the within-breed effects due to within family relationships have a very tiny effect on the between-breed level (as these do not confound our breed-specific estimates of the mean and variance of play behavior).

**Main analyses**

To account for within-breed variance in our analyses of human-directed behavior across the AKC groups, we used the “MCMCglmm” package (Hadfield 2010) and relied on a focal model that included AKC groups as main predictor (without intercept, see Table 1). Given the biological questions at hand, we relied on the following specifications: i) we set the initial values to take the estimated ancestral state score for human-directed play behavior; and ii) we constrained the parameter estimates for the fixed effects (i.e. group specific means) with an informative prior with a mean of 3 and variance of 1 (considering the possible range of scores); iii) the Markov chain had 1,050,000 iterations with an initial 50,000 burnin and subsequent 10 thinning intervals. To make an inference about the significance of the effect of AKC group categorization, we relied on the Deviance Information Criterion (DIC) of the model that was contrasted against the null model that only included the intercept. Lower DIC values mean relatively better fit to the data, thus we considered that the focal variable has a considerable effect if the fitted model had remarkably (deltaDIC > 10) smaller values than the respective null model. For the pairwise comparison of the posterior distribution of group-specific estimates of human-mediated play behavior, we estimated pMCMC, which is the probability that the difference between the corresponding posteriors encompasses zero (Makowski et al. 2019). Given that these Bayesian pMCMC values are not identical with the frequentist P values of statistical significance, and that we would have to adjust for a large number (21) of comparisions resulting in a considerable loss of power (see Nakagawa 2004), we avoided calculating adjusted Bayesian pMCMC values for multiple testing based on approaches from the frequentist framework, which is conceptually different.

In addition to the described model comparison concerning AKC groups as a reliable predictor in the models (providing a DIC difference of 13.6 unit, see results section in the main text), we created an alternative null model as a reference for the comparison by randomly shuffling the AKC groups among breeds. When we ran this model the DIC was 20.11 unit worse than the model that was based on the true groups, providing further support that the AKC group categorization is an important explanatory factor behind the variation in human-directed play behavior in the included breeds.

Before interpreting the outcomes of the MCMC modeling, we performed various diagnostics to verify that these returned with biologically interpretable results and not statistical artifacts. In particular, we have checked model convergence as was suggested in the tutorial of the *MCMCglmm* package (Hadfield 2010; 2012). This included checks for autocorrelation and the visual inspection of the trace of the sampled posterior as well as the density estimate of the posterior distribution. Furthermore, we also applied diagnostics tools such as Gelman and Rubin statistics (Gelman and Rubin 1992). We also verified if different runs with different lengths or with different priors (e.g. uninformative) return with the same output. These checks unanimously revealed that the model appropriately converged.

**Breed groups, breed group descriptions, and breeds included in each of the AKC breed groups. All information was derived from the AKC website:** <https://www.akc.org/expert-advice/lifestyle/7-akc-dog-breed-groups-explained/>. See supplemental excel sheet for the full dataset across the 132 breeds that were included in the analyses.

***Sporting group***

*AKC description*: “Breeds in the Sporting Group were bred to assist hunters in the capture and retrieval of feathered game. Retrievers, built for swimming, specialize on waterfowl, while the hunting grounds of setters, spaniels, and pointing breeds are grasslands where quail, pheasant, and other game birds nest. Many Sporting Group breeds possess thick, water-repellant coats resistant to harsh hunting conditions.”

*Included breeds*: American Water Spaniel, Barbet, Boykin Spaniel, Brittany, Chesapeake Bay retriever, Clumber Spaniel, Cocker Spaniel, Curly-Coated retriever, English Cocker Spaniel, English Setter, English Springer Spaniel, Field Spaniel, Flat-Coated Retriever, German Shorthaired Pointer, German Wirehaired Pointer, Golden Retriever, Gordon Setter, Irish Red and White Setter, Irish Setter, Irish Water Spaniel, Labrador Retriever, Lagotto Romagnolo, Nederlandse Kooikerhondje, Novia Scotia Duck Tolling Retriever, Pointer, Spinone Italiano, Sussex Spaniel, Vizsla, Weimaraner, Welsh Springer Spaniel, Wirehaired Pointing Griffon, Wirehaired Vizsla.

***Hound group***

*AKC description*: “All breeds in the Hound Group were bred to pursue warm-blooded quarry. The sleek, long-legged sighthounds use explosive speed and wide vision to chase swift prey, like jackrabbits and antelope, while tough, durable scenthounds rely on their powerful noses to trail anything from raccoons to escaped convicts. Members of the Hound Group possess strong prey drives and often will stop at nothing to catch their quarries.”

*Included breeds*: Afghan Hound, American English Coonhound, American Foxhound, Azawakh, Basenji, Basset Hound, Beagle, Black and Tan Coonhound, Bloodhound, Bluestick Coonhound, Borzoi, Cirneco dell’Etna, Dachshund, English Foxhound, Grand Basset Griffon Vendéen, Greyhound, Harrier, Ibizan Hound, Irish Wolfhound, Norwegian Elkhound, Otterhound, Petit Basset Griffon Vendéen, Pharaoh Hound, Plott Hound, Portuguese Podengo Pequeno, Redbone Coonhound, Rhodesian Ridgeback, Saluki, Scottish Deerhound, Sloughi, Treeing Walker Coonhound, Whippet.

***Working Group***

*AKC description*: “Breeds in the Working Group are dogkind’s punch-the-clock, blue-collar workers, and the group includes some of the world’s most ancient breeds. They were developed to assist humans in some capacity – including pulling sleds and carts, guarding flocks and homes, and protecting their families – and many of these breeds are still used as working dogs today. Breeds in the Working Group tend to be known for imposing stature, strength, and intelligence.”

*Included breeds*: Akita, Alaskan Malamute, Anatolian Shepherd Dog, Bernese Mountain Dog, Black Russian Terrier, Boerboel, Boxer, Bullmastiff, Cane Corso, Chinook, Doberman Pinscher, Dogo Argentino, Dogue de Bordeaux, German Pinscher, Giant Schnauzer, Great Dane, Great Pyrenees, Greater Swiss Mountain Dog, Komondor, Kuvasz, Leonberger, Mastiff, Neapolitan Mastiff, Newfoundland, Portugese Water Dog, Rottweiler, Saint Bernard, Samoyed, Siberian Husky, Standard Schnauzer, Tibetan Mastiff.

***Terrier Group***

*AKC description*: “The feisty, short-legged breeds in the Terrier Group were first bred to go underground in pursuit of rodents and other vermin. Long-legged terrier breeds dig out varmints rather than burrowing in after them, while the group’s “bully” breeds, created long ago for ghastly pursuits like bull-baiting, are popular companion dogs today. Breeds in the Terrier Group are excellent competitors in the sport of Earthdog.”

*Included breeds*: Airedale Terrier, American Hairless Terrier, American Staffordshire Terrier, Australian Terrier, Bedlington Terrier, Border Terrier, Bull Terrier, Cairn Terrier, Cesky Terrier, Dandie Dinmont Terrier, Glen of Imaal Terrier, Irish Terrier, Kerry Blue terrier, Lakeland terrier, Manchester Terrier (Standard), Miniature Bull Terrier, Miniature Schnauzer, Norfolk Terrier, Norwich Terrier, Parson Russell Terrier, Rat Terrier, Russell Terrier, Scottish Terrier, Sealyham Terrier, Skye Terrier, Smooth Fox Terrier, Soft Coated Wheaten Terrier, Staffordshire Bull Terrier, Welsh Terrier, West Highland White Terrier, Wire Fox Terrier.

***Toy Group***

*AKC description*: “The diminutive breeds of the Toy Group come in enough coat types and colors to satisfy nearly any preference, but all are small enough to fit comfortably in the lap of their adored humans. In a way, toy dogs are their own version of working dogs: they work hard at being attentive, affectionate companions. Breeds in the Toy Group are popular with city dwellers, as their small size makes them a good fit for smaller yards or apartments.”

*Included breeds*: Affenpinscher, Brussels Griffon, Cavalier King Charles Spaniel, Chihuahua, Chinese Crested, English Toy Spaniel, Havanese, Italian Greyhound, Japanese Chin, Maltese, Manchester Terrier (Toy), Miniature Pinscher, Papillon, Pekingese, Pomeranian, Poodle (Toy), Pug, Shih Tzu, Silky Terrier, Toy Fox terrier, Yorkshire Terrier.

***Non-Sporting group***

*AKC description*: “The breeds of the Non-Sporting Group have two things in common: wet noses and four legs. After that, there’s not much shared by this patchwork group of breeds whose job descriptions defy categorization in the six other groups, though they all have fascinating histories. Today, the varied breeds of the Non-Sporting Group are largely sought after as companion animals, as they were all developed to interact with people in some capacity.”

*Included breeds*: American Eskimo Dog, Bichon Frise, Boston terrier, Bulldog, Chinese Shar-Pei, Chow Chow, Coton de Tulear, Dalmatian, Finnish Spitz, French Bulldog, Keeshound, Lhasa Apso, Löwchen, Norwegian Lundehund, Poodle (Miniature), Poodle (Standard), Schipperke, Shiba Inu, Tibetan Spaniel, Tibetan Terrier, Xoloitzcuintli.

***Herding Group***

*AKC description*: “The Herding Group comprises breeds developed for moving livestock, including sheep, cattle, and even reindeer. Herding dogs work closely with their human shepherds, and their natural intelligence and responsiveness make them highly trainable. Today, some Herding breeds, such as the German Shepherd Dog, are commonly trained for police work. The high levels of energy found in Herding Group breeds mean finding them a job is recommended, lest they begin herding your children at home.”

*Included breeds*: Australian Cattle Dog, Australian Shepherd, Bearded Collie, Beauceron, Belgian Malinois, Belgian Sheepdog, Belgian Tervuren, Bergamasco Sheepdog, Berger Picard, Border Collie, Bouvier des Flandres, Briard, Canaan Dog, Cardigan Welsh Corgi, Collie, Entlebucher Mountain Dog, Finnish Lapphund, German Shepherd Dog, Icelandic Sheepdog, Miniature American Shepherd, Norwegian Buhund, Old English Sheepdog, Pembroke Welsh Corgi, Polish Lowland Sheepdog, Puli, Pumi, Pyrenean Shepherd, Shetland Sheepdog, Spanish Water Dog, Swedish Vallhund.

**References**

Garamszegi, L.Z., Temrin, H., Kubinyi, E., Miklósi, Á. & Kolm, N. 2020 The role of common ancestry and gene flow in the evolution of human-directed play behaviour in dogs. *J Evol Biol* **33**, 318-328

Gelman, A. & Rubin, D.B. 1992 Inference from iterative simulation using multiple sequences. *Stat. Sci.* **7**, 457-511.

Hadfield, J.D. 2010 MCMC methods for multi-response generalized linear mixed models: the MCMCglmm R package. *J. Stat. Softw*. **33**, 1-22.

Hadfield, J.D. 2012 MCMCglmm Course Notes. (University of Oxford). <https://cran.r-project.org/web/packages/MCMCglmm/vignettes/CourseNotes.pdf>

Makowski, D., Ben-Shachar, M. & Lüdecke, D. 2019 bayestestR: Describing Effects and their Uncertainty, Existence and Significance within the Bayesian Framework. *J. Open Source Softw*. **4**, 1541.

Nakagawa, S. 2004 A farewell to Bonferroni: the problems of low statistical power and publication bias. *Behav Ecol*, **15**, 1044-1045.

Turcsán, B., Kubinyi, E. & Miklósi, Á. 2011 Trainability and boldness traits differ between dog breed clusters based on conventional breed categories and genetic relatedness. Appl *Anim Behav Sci* **132**, 61-70.
